# Updating obesity management strategies: an audit of Italian specialists

**DOI:** 10.1007/s40519-022-01402-w

**Published:** 2022-05-17

**Authors:** Luca Busetto, Maria Grazia Carbonelli, Antonio Caretto, Annamaria Colao, Claudio Cricelli, Maurizio De Luca, Francesco Giorgino, Lucio Gnessi, Gerardo Medea, Giovanni Pappagallo, Ferruccio Santini, Paolo Sbraccia, Marco Antonio Zappa

**Affiliations:** 1 Departement of Medicine, University of Padova, Padova, Italy; 2 Dietology and Nutrition Unit, AO San Camillo Forlanini, Rome, Italy; 3 Presidente ADI (Italian Dietetic and Clinical Nutrition Association) Foundation, Rome, Italy; 4 Department of Clinical Medicine and Surgery, Università Federico II di Napoli, Naples, Italy; 5 Medico di Medicina Generale-Presidente SIMG (Italian Society of General Medicin), Firenze, Italy; 6 Department of General Surgery, Ospedale di Rovigo, Viale Tre Martiri 140, Rovigo, Italy; 7 Section of Internal Medicine, Endocrinology, Andrology and Metabolic Diseases, Department of Emergency and Organ Transplantation, University of Bari Aldo Moro, Bari, Italy; 8 Section of Medical Pathophysiology, Food Science and Endocrinology, Department of Experimental Medicine, Sapienza University of Rome, 00161 Rome, Italy; 9 Medico Di Medicina Generale, ATS Brescia; Responsabile nazionale ricerca e macroarea prevenzione della SIMG, Firenze, Italy; 10 School of Clinical Methodology, IRCCS “Sacre Heart-Don Calabria”, Negrar di Valpolicella, Italy; 11 Obesity and Lipodystrophy Center, Endocrinology Unit, University Hospital of Pisa, Pisa, Italy; 12 Department of Systems Medicine, Unit of Internal Medicine-Obesity Center, University of Rome Tor Vergata, Policlinico Tor Vergata, Rome, Italy; 13 Department of General Surgery, ASST Fatebenefratelli-Sacco Milano, Presidio ospedaliero Fatebenefratelli Milano; Presidente Nazionale Sicob (Italian Society of Obesity Surgery), Milan, Italy

**Keywords:** Obesity management, Adult obesity, Obesity comorbidities, Cardio-metabolic risk, Adipose tissue, Bariatric surgery

## Abstract

Obesity negatively affects physical and psychological health and increases health care costs. Although there is increasing interest in early diagnosis and timely intervention, there are several principles of care included in the current guidelines for clinical management of obesity that can potentially be updated and improved to address the “clinical inertia” and, consequently, to optimize the management of adult obesity. Using an online Delphi-based process, an Italian board of experts involved in the management of obesity discussed the usefulness of a pro-active approach to the care of patients with obesity, providing a consensus document with practical indications to identify risk factors for morbidity and death and raise awareness throughout the treatment continuum, including the early stages of the disease. In clinical practice, it seems inappropriate to delay an intervention that could avoid progression to a more severe level of obesity and/or prevent the onset of obesity-related comorbidities.

**Level of evidence** Level V, report of expert committee.

## Introduction

Despite numerous programs designed to encourage individuals and populations to lose weight, unfortunately, the global prevalence of obesity has increased in recent decades [1]. Obesity has become a major public health threat that increases health care costs and negatively affects physical and psychological health. People with obesity have an increased risk of hospitalization and mortality due to a state of chronic low-grade inflammation, an altered immune response to infections, and often multiple cardio-metabolic comorbidities [2–6]. Moreover, individuals living with obesity experience pervasive weight bias and stigma, which contribute to increased morbidity and mortality [7].

With its numerous compounding factors, obesity is increasingly being recognized as a treatable chronic disease. Although there is increasing interest in early diagnosis and timely intervention, several aspects of the current guidelines for clinical management of obesity are not at pace with a more advanced management of this disease. Strategies to address the “clinical inertia”, defined as the failure of health care providers to initiate or intensify therapy when indicated, should be better developed. According to the European guidelines for obesity management in adults [8], obesity surgery is foreseen in a patient with a body mass index (BMI) greater than 40 kg/m^2^, whereas in a patient with a BMI between 35 and 40 kg/m^2^, it should be considered only in the presence of comorbidities. Anti-obesity medications are indicated in patients with a BMI greater than 30 kg/m^2^, but in patients with a BMI greater than 27 kg/m^2^ they are indicated only in the presence of comorbidities. However, solid evidence shows that in individuals with prediabetes, the use of pharmacotherapy or bariatric surgery allows for a reduction in the risk of progression to diabetes regardless of BMI. Therefore, it seems inappropriate to delay an intervention that could avoid progression to a more severe level of obesity and/or prevent the onset of obesity-related comorbidities such as diabetes simply on the base of a BMI threshold. Moreover, current guidelines for the clinical management of obesity provide indications on what “can be done” but not on what “should be done”. This aspect stands in stark contrast to the indications of most guidelines for the management of other chronic conditions, including hypertension and dyslipidemia [9, 10], which clearly define the intensity of treatment based on the severity of the disease and related health risks. Finally, in the current guidelines for obesity management, the therapeutic intervention is not decided on the basis of a clear therapeutic target. For example, when the goal is to reduce the patient’s body weight, it should be possible to refer to a specific target. In addition, given a certain level of weight loss, it should be considered how to achieve that target. In summary, there are several principles of care in the current guidelines that can potentially be discussed and improved to optimize the management of adult obesity.

A multicenter workshop, based on a modified Delphi method, was developed by an Italian board of experts involved in the clinical management of obesity with the aim of improving the management of obesity as a chronic disease in clinical practice. This consensus document summarizes the evidence in favor of a structured approach to the management of patients with obesity, providing a practical framework to guide clinicians in identifying risk factors for morbidity and death and raise awareness throughout the treatment continuum, including the early stages of the disease.

## Methods

A consensus process was conducted using the Delphi methodology. This is a group-facilitative method designed to verify the convergence of an expert opinion panel in a given area of uncertainty within health-related research. The essence of a Delphi procedure is the exploration of expert views on a certain topic and the possibility for the experts to react to the input of the other experts over multiple rounds [11, 12]. Since the invention of the Delphi method in the 1950s, a commonly used variation of the Delphi method is the Estimate Talk-Estimate Delphi method that combines assembly of expert opinions on an anonymous basis during surveys with open exchange during workshops by a facilitator [13]. The process was developed over nearly 10 months through the following steps.

(i) Establishment of a Scientific Steering Committee of 11 experts who developed the statements to be ranked. First, members of the Steering Committee independently suggested those aspects of the disease worthy of clarification/deepening (items). Then, their consent on the list of items produced through a harmonization process by the facilitator was verified in a face-to-face meeting. The second Delphi round consisted of the generation of the statements of resolve for the critical issues by the Committee members. Subsequently, the members of the Steering Committee were again asked to reach consensus on the individual statements harmonized by the facilitator in a face-to-face meeting.
(ii) Selection of an expert panel of specialists. Forty-nine experts were selected from centers specialized in obesity management as representative of clinical practice in the field of obesity management in Italy. These experts were asked to rate their agreement or disagreement with each of the statements, independently and blindly. The survey was performed online on a secure survey website, using the online dedicated platform: “Obesitystepupteam”. Participants expressed their level of agreement with each statement using the RAND nine-point scale (ranging from 1 = completely disagree to 9 = completely agree) and a consensus was reached that a statement had to be considered appropriate if the median score was greater than or equal to 7.
(iii) Collection and analysis of the results. After the individual and anonymous online survey, the Scientific Steering Committee analyzed and discussed the collected results, and then defined several indications for the appropriate management of patients with obesity in clinical practice.

## Results

After the first Delphi round, the Scientific Steering Committee identified the following areas of obesity evaluation and management requiring an update (Table 1):

- Anthropometric assessments additional to the BMI.
- Measures of adiposity other than BMI (waist circumference [WC], waist-to-hip [W/H] ratio, etc.) to better stratify the cardio-metabolic risk.
- Evaluation of the obesity-related risk factors and comorbidities.
- Prediabetes as an obesity-related comorbidity requiring an intensified therapeutic approach.
- Use of anti-obesity medications in overweight patients (BMI 27–30 kg/m^2^) with prediabetes who cannot control body weight by lifestyle modifications alone.
- Use of bariatric surgery in patients with moderate obesity (BMI 35–40 kg/m^2^) and prediabetes who cannot control body weight with maximal medical therapy.
- Identification of specific treatment targets in patients with obesity based on the risk profile and clinical condition.
- Intensification of therapy in patients with obesity.
- BMI versus disease staging as the main guiding criteria for choosing the therapy level in patients with obesity.
- Intensification of therapy in patients with severe obesity and/or newly identified advanced stage.

**Table 1:** Statements approved by the Scientific Steering Committee and rated by the expert panel

Item	Statement	Expert panel ranking
1. Anthropometric assessments additional to the BMI	If the patient’s clinical features make the clinical significance of BMI questionable, the anthropometric assessment of the patient should include measurement of abdominal adipose tissue accumulation (waist circumference) and instrumental evaluation of fat mass, i.e., dual-energy X-ray absorptiometry or bioimpedance analysis	Median: 9 Consent: 87% Uncertain: 10% Dissent: 3%
2. Measures of adiposity other than BMI (WC, W/H ratio, etc.) to better stratify the cardio-metabolic risk	Cardio-metabolic risk stratification in patients with obesity should be based on systematic measurement of adipose tissue distribution in addition to the BMI	Median: 8 Consent: 92% Uncertain: 8% Dissent: 0%
3. Evaluation of the obesity-related risk factors and comorbidities	Assessment of the presence of medical comorbidities, psychological status, and severity of disability should be performed systematically, using clinical, biochemical, and instrumental parameters with an advantageous cost-effectiveness ratio	Median: 9 Consent: 95% Uncertain: 5% Dissent: 0%
4. Prediabetes as an obesity-related comorbidity requiring an intensified therapeutic approach	Impaired fasting glucose and especially impaired glucose tolerance are risk factors for developing type 2 diabetes. These conditions can, therefore, make a patient eligible for a level of therapy that requires the presence of at least one comorbidity, in addition to a given BMI value	Median: 8 Consent: 87% Uncertain: 5% Dissent: 8%
5. Use of anti-obesity medications in overweight patient (BMI 27–30 kg/m^2^) with prediabetes who cannot control body weight by lifestyle modifications alone	In patients with BMI 27–30 kg/m^2^ who cannot control body weight with lifestyle modification only, the presence of prediabetes is a sufficient criterion to consider anti-obesity pharmacologic therapy	Median: 8 Consent: 82% Uncertain: 18% Dissent: 0%
6. Use of bariatric surgery in patients with moderate obesity (BMI 35–40 kg/m^2^) and prediabetes, who cannot control body weight with maximal medical therapy	In patients with BMI 35–40 kg/m^2^ who cannot control body weight with maximal medical therapy, a prediabetes condition is a sufficient criterion to consider bariatric surgery, also based on the age and overall cardio-metabolic risk profile of the patient	Median: 8 Consent: 82% Uncertain: 15% Dissent: 3%
7. Identification of specific treatment targets in patients with obesity based on the risk profile and clinical condition	Weight loss is only one of the aspects involved in the entire psycho-physical complexity of patients with obesity. In line with the principles of precision medicine, the therapeutic goal must be individualized, realistic, shared with the patient and should take into account the complexity of the clinical situation associated with obesity, as well as the history of the patient’s weight and dietary attempts made. The extent of weight loss should be commensurate with the specific medical comorbidities, psychological status and severity of disability	Median: 9 Consent: 92% Uncertain: 8% Dissent: 0%
8. Intensification of therapy in patients with obesity	Intensification of therapy in patients with obesity should be started early if the patient is at risk of comorbidities and/or when there is evidence for a preventive role of weight loss in the occurrence of specific comorbidities	Median: 9 Consent: 92% Uncertain: 3% Dissent: 5%
9. BMI versus disease staging as the main guiding criteria for choosing the therapy level in patients with obesity	In patients with obesity, the main guiding criterion for the choice of therapy level (lifestyle modification/anti-obesity pharmacologic therapy/bariatric surgery) should be not only the BMI value but also the disease stage, based on assessment of the medical comorbidities, psychological status and severity of disability	Median: 9 Consent: 92% Uncertain: 5% Dissent: 3%
10. Intensification of therapy in patients with severe obesity and/or newly identified advanced stage	In patients with severe obesity and/or advanced stage, it is advisable to immediately consider the pharmacologic or surgical therapy according to specific indications/contraindications, in addition to lifestyle modifications	Median: 9 Consent: 85% Uncertain: 10% Dissent: 5%

BMI, body mass index; W/H ratio, waist-to-hip ratio; WC, waist circumference

As a result of the second Delphi round, 10 statements were approved by the Scientific Steering Committee and submitted to the independent experts for their ratings (Table 1).

Thirty-nine of the 49 experts (79.6%) rated the agreement or disagreement for each of the 10 statements regarding different issues related to obesity management. The names of the 39 experts providing a rating are listed in the Appendix. The median experts rating and the percentages of experts consenting, being uncertain, or dissenting for each statement are reported in Table 1. The median expert rating was 9 for 6 statements and 8 for the remaining 4 statements. All statements received a marginal percentage of disagreement. No further rounds of rating were deemed to be necessary and the Scientific Steering Committee discussed and finalized the statements.

## Discussion

The audit of Italian experts involved in the field of obesity, conducted using a Delphi methodology and presented in this paper, identified and agreed on 10 statements suggesting an update of obesity management as a chronic disease in clinical practice.

### Anthropometric assessments additional to the BMI

**Statement 1**: If the patient’s clinical features make the clinical significance of BMI questionable, the anthropometric assessment of the patient should include measurement of abdominal adipose tissue accumulation (waist circumference) and instrumental evaluation of fat mass, i.e., dual-energy X-ray absorptiometry (DXA) or bioimpedance analysis (BIA) (Expert panel median consensus estimate: 9).

It became clear in recent years that diagnosis of obesity based solely on BMI lacked the information needed for adequate clinical decision-making in obesity management. Despite the good correlation between BMI and percentage of body fat observed in large epidemiological samples, the accuracy of BMI to diagnose excessive fat accumulation was limited, particularly for individuals in the intermediate BMI ranges. In individuals with a normal or mild increased BMI, the diagnostic performance of BMI is limited due to its inability to discriminate between fat and lean mass [14]. It was agreed that, for an appropriate anthropometric assessment of adult patients, other measurements of adiposity may be considered at the clinician’s discretion if BMI and physical examination results are equivocal. In particular, WC and evaluation of fat mass, performed by DXA and/or BIA, provide accurate information in addition to BMI when evaluating patients at risk of adiposity-related medical complications [15]. Therefore, centers that are unable to perform instrumental evaluations should refer patients who require these evaluations to centers equipped to perform them.

### Measures of adiposity other than BMI (waist circumference, waist-to-hip ratio, etc.) to better stratify the cardio-metabolic risk

**Statement 2:** Cardio-metabolic risk stratification in patients with obesity should be based on systematic measurement of adipose tissue distribution in addition to BMI (Expert panel median consensus estimate: 8).

In clinical practice, the measurement of adipose tissue distribution should be performed on all patients with obesity. Body fatness is estimated by BMI and the accumulation of intra-abdominal fat (a marker for higher metabolic and cardiovascular disease [CVD] risk) can be assessed by WC or W/H ratio [8, 16, 17]. In addition to BMI, WC and W/H ratio were shown to be independent variables in predicting the risk of developing hypertension and impaired glucose tolerance in both sexes [18]. WC is measured in the horizontal plane midway in the distance between the superior iliac crest and the lower margin of the last rib. European guidelines for adult obesity management define central obesity (also known as visceral, android, apple-shaped, or upper body obesity) in Europids as a WC ≥ 94 cm in men and ≥ 80 cm in non-pregnant women [8].

### Evaluation of obesity-related risk factors and comorbidities

**Statement 3**: Assessment of the presence of medical comorbidities, psychological status, and severity of disability should be performed systematically, using clinical, biochemical, and instrumental parameters with an advantageous cost-effectiveness ratio (Expert panel median consensus estimate: 9).

Excessive adiposity can predispose individuals to the development of several medical comorbidities, such as type 2 diabetes (T2D), chronic kidney disease, gallbladder disease, nonalcoholic fatty liver disease, gout, obstructive sleep apnea, and osteoarthritis [19]. Excess and ectopic body fat are relevant sources of adipocytokines and inflammatory mediators that can alter glucose and fat metabolism, leading to increased cardio-metabolic and cancer risks. Obesity increases the risk of colon, kidney, esophageal, endometrial, and postmenopausal breast cancers [19]. A multidimensional assessment, which takes into consideration various aspects including the psychological one, is a fundamental step in the management of patients with obesity. It is recommended to assess WC, neck circumference, fasting glucose or glycated hemoglobin values, blood pressure, and lipid profile, including total cholesterol, triglycerides, and high-density lipoprotein-cholesterol, to determine the cardio-metabolic risk, and when indicated, alanine aminotransferase to screen for nonalcoholic fatty liver disease [15, 19]. The European Society of Endocrinology Clinical Guideline defines the endocrine workup in patients with obesity [20]. It is recommended that all patients with obesity are tested for thyroid function. The European experts recommended that testing for hypothyroidism is based on the level of thyroid-stimulating hormone level; if this is increased, free thyroxine and antibodies (anti-thyroid peroxidase) should be measured. For hypercortisolism, male hypogonadism, and female gonadal dysfunction, hormonal testing is only recommended in the case of clinical suspicion of an underlying endocrine disorder [20].

The Edmonton Obesity Staging System (EOSS) considers metabolic, physical, and psychological parameters to guide clinical decisions and to determine the optimal obesity treatment [21]. Recently, integration of EOSS with a new functional parameter obtained by cardiopulmonary exercise testing, i.e., cardiorespiratory fitness (CRF), expressed as weight-adjusted peak oxygen consumption, has been suggested. This integration allows a severity index that considers not only clinical parameters but also their functional impairment to be assigned to each patient [22].

### Prediabetes as an obesity-related comorbidity requiring an intensified therapeutic approach

**Statement 4**: Impaired fasting glucose and especially impaired glucose tolerance are risk factors for developing type 2 diabetes. These conditions can, therefore, make a patient eligible for a level of therapy that requires the presence of at least one comorbidity, in addition to a given BMI value (Expert panel median consensus estimate: 8).

Conditions at risk for developing T2D, such as impaired fasting glucose (IFG) and especially impaired glucose tolerance (IGT), deserve particular attention and should promote more aggressive treatment. The criteria for defining IFG and IGT remain those recommended by the American Diabetes Association (ADA) and adopted by the Diabetes Physicians Association (AMD) and the Italian Society of Diabetology (SID) [23, 24].

### Use of anti-obesity medications in overweight patients (BMI 27–30 kg/m^2^) with prediabetes who cannot control body weight by lifestyle modifications alone

**Statement 5:** In patients with BMI 27–30 kg/m^2^ who cannot control body weight with lifestyle modifications only, the presence of prediabetes is a sufficient criterion to consider anti-obesity pharmacologic therapy (Expert panel median consensus estimate: 8).

For the overweight patient with prediabetes, a more daring approach should be envisaged to overcome therapeutic inertia in the management of obesity. Although not provided for in current obesity guidelines, this approach is supported by important scientific evidence. Medication-assisted weight loss should be considered in patients at risk for future T2D and should be used when needed to achieve at least 10% weight loss in conjunction with lifestyle interventions [15]. Three years of treatment with liraglutide in adults with prediabetes and a BMI of at least 30 kg/m^2^, or at least 27 kg/m^2^ with comorbidities, might provide health benefits in terms of reduced risk of T2D [25]. A systematic review was conducted to support the U.S. Preventive Services Task Force in updating its 2012 recommendation on screening for and treatment of adults with obesity. Data analysis showed that behavior-based weight-loss interventions with or without weight-loss medications resulted in a decreased risk of developing T2D, particularly among those with prediabetes [26].

### Use of bariatric surgery in patients with moderate obesity (BMI 35–40 kg/m^2^) and prediabetes who cannot control body weight with maximal medical therapy

**Statement 6:** In patients with BMI 35–40 kg/m^2^ who cannot control their body weight with maximal medical therapy, a prediabetes condition should be considered as a sufficient criterion for proposing bariatric surgery, also based on the age and overall cardio-metabolic risk profile of the patient (Expert panel median consensus estimate: 8).

Patients with prediabetes or T2D require extra care [27]. Weight loss induced by surgery has proven to be highly efficacious in treating obesity and its comorbidities. The awareness that some diseases related to obesity, such as T2D, obstructive sleep apnea syndrome (OSAS), and others, has a considerable effect on the prognosis of patients and demonstration of the effectiveness of surgery in improving or resolving these morbid conditions have led to a radical extension of the indications in this field [28]. The indication for surgery should not be based solely on BMI. In general, a shift in the paradigm should occur focusing more on the disease treatment and overall level of disability rather than simply on body weight.

The International Federation for the Surgery of Obesity and Metabolic Disorders (IFSO) reached a consensus on indicating bariatric surgery for obesity and weight-related diseases stating that surgery for obesity and weight-related diseases may be an effective therapeutic option for the management of obese patients with T2D, demonstrating good results in terms of glycemic control, glycosylated hemoglobin, and diabetes medications [28]. Furthermore, weight loss achieved with surgery improves components of the metabolic syndrome. Surgery for obesity and weight-related diseases reduces the risk of CVD in terms of atherosclerosis, myocardial infarction, stroke, and death [29]. Specifically, results from the Swedish Obese Subjects (SOS) study clearly demonstrated that the sustained weight loss produced by surgery is associated to a highly significant reduction in the incidence of new cases of T2D in patients with prediabetes at enrollment [27].

### Identification of specific treatment targets in patients with obesity based on the risk profile and clinical condition

**Statement 7:** Weight loss is only one of the aspects involved in the entire psycho-physical complexity of patients with obesity. In line with the principles of precision medicine, the therapeutic goal must be individualized, realistic, shared with the patient, and should take into account the complexity of the clinical situation associated with obesity, as well as the history of the patient's weight and dietary attempts made. The extent of weight loss should be commensurate with the specific medical comorbidities, psychological status and severity of disability (Expert panel median consensus estimate: 9).

Obesity has become a major public health issue that has a negative impact on physical and psychological health. Individuals with obesity experience pervasive weight bias and stigma, which contribute to increased morbidity and mortality independently of weight or BMI [2, 30]. Health care providers should talk to patients with obesity and agree on realistic expectations and person-centered treatments. People living with obesity should receive tailored care plans that address the root cause of their obesity, meet individual values and preferences, and offer support for behavior modification (e.g., nutrition, physical activity) and adjunctive interventions, which may include psychological, pharmacologic, and surgical therapies [19]. Over the years, relevant advances have occurred in all modalities used to treat obesity: lifestyle interventions, pharmacotherapy, and weight-loss procedures, including bariatric surgery [19]. Specific pharmacotherapies are recommended for use in different clinical settings based on efficacy, safety profile, warnings and contraindications, organ clearance, mechanisms of action, and available evidence for use of the therapy under these specific conditions [19].

### Intensification of therapy in patients with obesity

**Statement 8:** Intensification of therapy in patients with obesity should be started early if the patient is at risk of comorbidities and/or when there is evidence for a preventive role of weight loss in the occurrence of specific comorbidities (Expert panel median consensus estimate: 9).

Adipose tissue accumulation and dysfunction clearly play a role in the genesis of several obesity-related diseases, such as T2D, CVD, some cancers, kidney disease, OSAS, gout, osteoarthritis, and hepatobiliary diseases, among others. For several obesity-related diseases, there is a curvilinear increase in risk as a function of weight [6]. Evidence from randomized controlled trials revealed that weight-loss interventions, such as pharmacotherapy and lifestyle changes, can reduce the risk of obesity-related conditions including OSAS [31] and delay the onset of T2D [25, 32]. Observational data from the Osteoarthritis Initiative (OAI) showed that greater weight loss is associated with less cartilage degradation [33]. Data from the Diabetes Prevention Program [34] and the Action for Health in Diabetes (Look AHEAD) study [35] demonstrated that weight loss has a beneficial effect on diabetes and CVD outcomes (among those losing > 10% of their body weight in the first year), respectively. Furthermore, long-term follow-up evidence from the SOS study indicated that bariatric surgery resulted in significant reductions in cardiovascular mortality and occurrence of fatal and non-fatal cardiovascular events [36], as well as in the incidence of T2D [37]. A recent analysis estimated how the risks of 10 obesity-related outcomes change in response to weight loss of 13%. The results of this study showed a risk reduction after weight loss for T2D (41%), OSAS (40%), hypertension (22%), dyslipidemia (19%), and asthma (18%). Furthermore, weight loss was associated with additional benefits, with lower risk of T2D, chronic kidney disease, hypertension, and dyslipidemia compared with maintaining the corresponding stable lower BMI throughout the study [38]. Available evidence emphasizes the importance of obtaining predefined levels of weight loss in patients with obesity with or at risk for obesity-related comorbidities and suggests prompt intensification of treatment when these levels are not reached with a first-line treatment strategy.

### BMI versus disease staging as the main guiding criteria for choosing the therapy level in patients with obesity

**Statement 9**: In patients with obesity, the main guiding criteria for the choice of therapy level (lifestyle modification/anti-obesity pharmacologic therapy/bariatric surgery) should be not only the BMI value but also the disease stage, based on assessment of the medical comorbidities, psychological status, and severity of disability (Expert panel median consensus estimate: 9).

The development of comorbidities or complications of obesity, which appear in most patients during progression of the disease, often does not correlate linearly with BMI. Many variables contribute to their occurrence regardless of the degree of obesity measured with the BMI: disease duration, age, sex, distribution of adipose tissue depots, genetic background, degree of mechanical disability, among others. The therapeutic strategy should take into account the severity of obesity (BMI), together with the presence and severity of complications and age, to better choose the interventions, from lifestyle modifications to bariatric surgery [17, 39]. Treatment plans should be individualized. Suggested interventions should be appropriate for obtaining the required degree of weight loss to address the obesity-related comorbidities at the specific stage of severity [15, 19].

### Intensification of therapy in patients with severe obesity and/or newly identified advanced stage

**Statement 10:** In patients with severe obesity and/or advanced stage, it is advisable to immediately consider the pharmacologic or surgical therapy according to specific indications/contraindications, in addition to lifestyle modifications (Expert panel median consensus estimate: 9).

Current guidelines for clinical management of obesity [8] usually suggest a stepwise therapeutic strategy in which patients should be treated with lifestyle modifications alone as the first line, and anti-obesity medications and bariatric surgery can be indicated for resistant cases. This approach is different compared with the indications in most guidelines for the management of chronic conditions, including hypertension and dyslipidemia [9, 10], which clearly define that the intensity of the initial treatment should be based on the severity of the disease. Escalation to anti-obesity medications or bariatric surgery could be considered in a patient with obesity in whom the presence of comorbidities and the personal history suggest a low chance of reaching adequate levels of weight loss with lifestyle modifications alone.

## Conclusions

This consensus document summarizes the evidence in favor of a pro-active approach to the care of patients with obesity, providing a practical framework to guide physicians in identifying risks for morbidity and premature death and raise clinical awareness in the early stages of the disease process.

The widespread, yet unproven, assumption that excess body weight in individuals with obesity derives primarily from a lack of self-discipline and personal responsibility has a major impact on how obesity prevention and management are implemented. The efficacy of anti-obesity interventions based solely on patients’ education and lifestyle change could be overestimated, promoting therapeutic inertia in clinical guidelines and health care professionals and limiting or delaying the adoption of more effective therapeutic strategies, including pharmacologic and surgical therapies [40]. Increased understanding of the pathophysiologic mechanisms causing and maintaining obesity in the long-term highlights that body weight regulation is not entirely under volitional control, and that biological, genetic, and environmental factors contribute critically to obesity. However, there is a gap between scientific evidence and misconceptions in the public narrative [7]. Voluntary attempts to eat less and exercise more result in only modest effects on body weight loss in most individuals with severe obesity [41, 42]. When fat mass decreases, the body responds with reduced resting energy expenditure, and changes in signals that increase hunger and reduce satiety [43]. These compensatory metabolic and biological adaptations promote weight regain and persist for as long as patients are in a reduced-energy state, even if they gain some weight back [44].

The comprehension of obesity as a chronic disease linked to a profound disruption of the physiologic mechanisms regulating eating behavior and energy balance should also influence the way in which obesity is managed. Clinicians should shift from a stepwise approach largely focused on BMI alone to a more advanced approach in which the intensity of therapeutic intervention is based on a complete evaluation of the obesity status and the intensity of treatment should be promptly adjusted to the patient’s needs and therapeutic targets. We hope that the audit of Italian obesity experts presented in this paper could help to modify clinical practices in obesity management in this direction, preventing therapeutic inertia [40].

## Strength and limits

The strength of this consensus is the large number of Italian obesity specialists involved in the formulation of the statements. The national focus limits the applicability of the statements to other countries but reinforces the possibility of their application in the Italian context.

## What is already known on this subject?

The assumption that obesity derives primarily from a lack of self-discipline and personal responsibility has a major impact on how obesity prevention and management are implemented. The efficacy of anti-obesity interventions based solely on patients’ education and lifestyle change is overestimated, promoting therapeutic inertia and limiting or delaying the adoption of more effective therapeutic strategies.

## What this study adds?

The comprehension of obesity as a chronic disease linked to a profound disruption of the physiologic mechanisms regulating eating behavior and energy balance should also influence the way in which obesity is managed. Clinicians should shift from a stepwise approach largely focused on BMI alone to a more advanced approach in which the intensity of therapeutic intervention is based on a complete evaluation of the obesity status, and the intensity of treatment should be promptly adjusted to the patient’s needs and therapeutic targets.

## Appendix: Expert panel

**Carmela Bagnato** (ASM Matera)

**Luigi Barrea** (Università degli Studi di Napoli Federico II)

**Maurizio Battino** (Università Politecnica delle Marche, Ancona)

**Rocco Barazzoni** (Ospedale Cattinara—Università di Trieste)

**Silvia Bettini** (Università degli Studi di Padova)

**Simona Bo** (Università di Torino, Città della Salute e della Scienza di Torino)

**Giovanni Casella** (Università di Roma La Sapienza)

**Stefano Celotto** (AAS3 Alto Friuli—Collina—Medio Friuli, Udine)

**Cristiano Crisafulli** (ASP Catania, Distretto di Acireale)

**Monica D’Adamo** (Università di Roma Tor Vergata)

**Riccardo Dalle Grave** (Casa di Cura Villa Garda, Garda, Verona)

**Nicola Di Lorenzo** (Università di Roma Tor Vergata)

**Diego Foschi** (Ospedale L Sacco, Università degli Studi di Milano)

**Lucia Frittitta** (Ospedale Garibaldi, Catania)

**Simona Frontoni** (Ospedale Fatebenefratelli-Isola Tiberina, Università di Roma Tor Vergata)

**Alfredo Genco** (Università di Roma La Sapienza)

**Ilenia Grandone** (Ospedale Santa Maria Terni)

**Ignazio Grattagliano** (Università degli Studi di Bari Aldo Moro)

**Chiara Graziadio** (AOU Federico II Napoli)

**Valeria Guglielmi** (Università di Roma Tor Vergata)

**Valeria Lagattolla** (ASL Bari)

**Carla Lubrano** (Università di Roma La Sapienza)

**Lucio Lucchin** (Azienda Sanitaria dell’Alto Adige Comprensorio di Bolzano)

**Giovanni Merola** (Ospedale San Giovanni di Dio, Frattamaggiore, Napoli)

**Fausta Micanti** (Università degli Studi di Napoli Federico II)

**Fabrizio Muratori** (Ospedale Sant'Anna ASST Lariana, Como)

**Giovanna Muscogiuri** (Università degli Studi di Napoli Federico II)

**Giuseppe Navarra** (AOU Policlinico di Messina)

**Barbara Neri** (Azienda Ospedaliera S Camillo-Forlanini Roma)

**Barbara Paolini** (Ospedale S Maria Alle Scotte, Università di Siena)

**Maria Letizia Petroni** (Alma Mater Studiorum Università di Bologna, IRCCS Policlinico Sant’Orsola, Bologna)

**Giacomo Piatto** (Ospedale di Montebelluna, ULSS 2 Marca Trevigiana, Treviso)

**Stefano Pintus** (ARNAS GBrotzu, Cagliari)

**Angelo Michele Schettino** (Clinica S Lorenzino Cesena)

**Roberto Serra** (Azienda Ospedaliera Università di Padova)

**Roberto Vettor** (Università degli Studi di Padova)

** Luisella Vigna** (Fondazione IRCCS Ca’ Granda, Ospedale Maggiore Policlinico, Milano)

**Mikiko Watanabe** (Università di Roma La Sapienza)

**Maria Grazia Zenti** (Azienda Ospedaliera di Verona)
